# Aqueous Blackcurrant Extract Improves Insulin Sensitivity and Secretion and Modulates the Gut Microbiome in Non-Obese Type 2 Diabetic Rats

**DOI:** 10.3390/antiox10050756

**Published:** 2021-05-10

**Authors:** Hye-Jeong Yang, Ting Zhang, Xuan-Gao Wu, Min-Jung Kim, Young-Ho Kim, Eun-Suk Yang, Yeong-Seok Yoon, Sunmin Park

**Affiliations:** 1 Research Division of Food Functionality, Korean Food Research Institutes, Wanjoo 55365, Korea; yhj@kfri.re.kr (H.-J.Y.); kmj@kfri.re.kr (M.-J.K.); 2 Department of Bio-Convergence System, Hoseo University, Asan 31499, Korea; zhangting92925@gmail.com (T.Z.); niyani0@naver.com (X.-G.W.); 3 Hanter Co., Ltd., Jeongeup 56204, Korea; kimyh@tiumter.com (Y.-H.K.); yoonys@tiumter.com (Y.-S.Y.); 4 Jiwon Co., Ltd., Jeongeup 56212, Korea; yanges@tiumter.com; 5 Department of Food and Nutrition, Obesity/Diabetes Research Center, Hoseo University, Asan 31499, Korea

**Keywords:** blackcurrants, insulin sensitivity, insulin secretion, inflammation, β-cell mass, gut microbiota

## Abstract

This study was undertaken to determine whether aqueous blackcurrant extracts (BC) improve glucose metabolism and gut microbiomes in non-obese type 2 diabetic animals fed a high-fat diet and to identify the mechanism involved. Partially pancreatectomized male Sprague–Dawley rats were provided a high-fat diet containing 0% (control), 0.2% (L-BC; low dosage), 0.6% (M-BC; medium dosage), and 1.8% (H-BC; high dosage) blackcurrant extracts; 0.2% metformin (positive-C); plus 1.8%, 1.6%, 1.2%, 0%, and 1.6% dextrin, specifically indigestible dextrin, daily for 8 weeks. Daily blackcurrant extract intakes were equivalent to 100, 300, and 900 mg/kg body weight (bw). After a 2 g glucose or maltose/kg bw challenge, serum glucose and insulin concentrations during peak and final states were obviously lower in the M-BC and H-BC groups than in the control group (*p* < 0.05). Intraperitoneal insulin tolerance testing showed that M-BC and H-BC improved insulin resistance. Hepatic triglyceride deposition, TNF-α expression, and malondialdehyde contents were lower in the M-BC and H-BC groups than in the control group. Improvements in insulin resistance in the M-BC and H-BC groups were associated with reduced inflammation and oxidative stress (*p* < 0.05). Hyperglycemic clamp testing showed that insulin secretion capacity increased in the acute phase (2 to 10 min) in the M-BC and H-BC groups and that insulin sensitivity in the hyperglycemic state was greater in these groups than in the control group (*p* < 0.05). Pancreatic β-cell mass was greater in the M-BC, H-BC, and positive-C groups than in the control group. Furthermore, β-cell proliferation appeared to be elevated and apoptosis was suppressed in these three groups (*p* < 0.05). Serum propionate and butyrate concentrations were higher in the M-BC and H-BC groups than in the control group. BC dose-dependently increased α-diversity of the gut microbiota and predicted the enhancement of oxidative phosphorylation-related microbiome genes and downregulation of carbohydrate digestion and absorption-related genes, as determined by PICRUSt2 analysis. In conclusion, BC enhanced insulin sensitivity and glucose-stimulated insulin secretion, which improved glucose homeostasis, and these improvements were associated with an incremental increase of the α-diversity of gut microbiota and suppressed inflammation and oxidative stress.

## 1. Introduction

The global prevalence of type 2 diabetes (T2DM) increased from 4.7% in 1980 to 8.5% in 2014 among adults (>18 years old) [1], and older adults were reported in 2017 to show higher prevalence as follows: 15% among 50- to 60-year-olds and 22% among those aged >70 [2]. The prevalence of T2DM is equal in men and women [2] but is influenced by ethnicity; for example, it is 7.5% among non-Hispanic whites, 9.2% among Asian Americans, 12.5% among Hispanics, 11.7% among non-Hispanic blacks, and 14.7% among Native Americans. Furthermore, the prevalence of T2DM is increasing more rapidly among Asians than among non-Asians, even though Asians are less obese [3]. The global all-cause mortality rate attributed to T2DM is 14.5% among adults [4], and diabetes and its cardiovascular and kidney complications are primary causes of mortality in many countries [4].

Blood glucose concentrations are regulated by the balance between insulin resistance and insulin secretion. When insulin resistance is elevated, insulin secretion must be increased, but if insulin secretion cannot compensate for insulin resistance, T2DM can be induced [3]. Asians have lower insulin secretion capacities and β-cell masses than Caucasians and thus are more susceptible to T2DM [3]. These ethnic differences are related to genetic and gut microbiome differences [5,6,7]. In concert with improvements in economic status, Asians now adopt more sedentary lifestyles and eat more refined and fatty foods. These lifestyle changes increase insulin resistance and are considered to be responsible for the marked increases in the incidences of T2DM observed among Asians.

T2DM progression is also associated with the gut microbiome, but T2DM-associated changes in gut microbiome composition remain controversial. Gut microbiota alter the intestinal contents of short-chain fatty acids (SCFAs), trimethylamine N-oxide (TMAO), lipopolysaccharide (LPS), aromatic amino acids, and other metabolites, as well as altering glucose metabolism [8]. *Prevotella* in the gut may be associated with T2DM but reported results are inconsistent in experimental animals and humans [9,10]. Metformin and berberine reduce serum LPS concentrations, alleviate intestinal inflammation, ameliorate the intestinal barrier structure [11], and increase the abundance of SCFA-producing bacteria, such as *Butyricimonas*, *Coprococcus*, and *Ruminococcus*, and beneficial bacteria, such as *Lactobacillus* and *Akkermansia* [11]. However, they reduce the relative abundances of *Prevotella* and *Proteus* [11]. Despite the lack of a comprehensive understanding of the effects of gut microbiota on whole-body energy metabolism, it is generally accepted that gut microbiota composition plays a critical role in the progression and treatment of T2DM.

Several medications are available for T2DM but unfortunately, they have adverse effects [12]. T2DM prevention is viewed as a priority by the World Health Organization and the United Nations and, in 2018, the Berlin Declaration called for global action to address the T2DM pandemic [13]. Individuals at high risk of developing T2DM are recommended to adopt lifestyle modifications, and functional foods are suggested to prevent and alleviate T2DM. A daily intake of anthocyanins >300 mg, including cyanidin and malvidin, for over eight weeks has been reported to reduce insulin resistance in overweight and obese T2DM patients, and black carrot extract intake (500 mg/kg body weight (bw)) for 12 weeks has been reported to reduce insulin resistance [14]. Anthocyanins protect β cells in part by reducing oxidative stress in estrogen-deficient rats [15,16]. Blackcurrant berries (*Ribes nigrum* L.) have high antioxidant and anti-inflammatory properties and improve blood glucose regulation [17,18]. Their major bioactive components are cyanidin 3-*O*-rutinoside and delphinidin 3-*O*-rutinoside, and they have also been reported to have anti-inflammatory, immunomodulatory, antioxidant, and antimicrobial properties [17]. They also demonstrate α-amylase and α-glucosidase inhibitory activities and stimulate AMPK activity to improve glucose metabolism [15,19]. Previous studies have shown that intake of 600 mg of anthocyanin-rich blackcurrant 30 min before a high carbohydrate meal reduces serum glucose concentration and elevates gastric inhibitory peptide (GIP) and glucagon-like peptide-1 (GLP-1) in post-menopausal women and men [20]. In overweight and obese T2DM animals, a 1.1% blackcurrant extract diet intake over seven weeks lowered serum glucose concentrations in fasting and 2 h postprandial states and improved insulin sensitivity [21]. Asian T2DM patients are leaner than their Caucasian counterparts and thus we suspected that the effect of blackcurrant supplementation on T2DM in Asians might differ [3]. Furthermore, no previous study has examined the effect of BC intake on the functionality of gut microbiota. Accordingly, we investigated whether aqueous blackcurrant extracts improve glucose homeostasis and gut microbiome compositions in partially pancreatectomized rats, a T2DM animal model for Asians, fed a high-fat diet.

## 2. Materials and Methods

### 2.1. Preparation of Powdered Aqueous Blackcurrant Extract and Analysis of Its Ingredients

Blackcurrants (Heukdan) were washed and ground with a blender, then extracted with distilled water (1:10, *w*/*v*) at 80 °C for 2 h and filtered through 50 μm filter paper. Filtrates were concentrated at 60 °C under vacuum conditions and concentrates were lyophilized. The yield of the powdered blackcurrant extract obtained was 24.8%. Lyophilized water extracts of blackcurrants were dissolved in methanol and passed through a syringe filter to remove undissolved contents. Cyanidin 3-*O*-rutinoside and delphinidin 3-*O*-rutinoside levels, as index compounds, were measured using high-performance liquid chromatography (HPLC; JASCO, Easton, MD, USA) using a YMC-Pak ODS-AM 303 column (5.0 μm, 250 × 4.6 mm) and a UV detector at 520 nm. The mobile phase solvents were 1% formic acid in water (A) and 1% formic acid in acetonitrile (B). These compounds were separated with the gradient elution protocol (A:B = 9:1 for 0 to 34 min, A:B = 6:4 for 34 to 35 min, and A:B = 9:1 for 36 to 46 min) at a flow rate of 0.7 mL/min and an in-column temperature of 30 °C. Delphinidin-3-rutinoside and cyanidin-3-rutinoside were used as standards for quantification purposes. Total anthocyanin contents were measured according to color differences of the samples at pH 1.0 and pH 4.5 resulting from chemical structure changes, which is called the pH differential method [22]. Cyanidin-3—glucoside was used as a reference compound.

### 2.2. Animals and Ethics

Eight-week-old male Sprague–Dawley rats (weight: 240 ± 21 g) were housed individually in stainless steel cages in a controlled environment (23 °C; 12 h light/dark cycle). All surgical and experimental procedures were performed according to the guidelines issued by the Animal Care and Use Review Committee of Hoseo University, Korea (HSIACUC-19-036). The rats underwent 60% pancreatectomy, which was performed as follows. After inducing anesthesia with an intramuscular injection of a ketamine and xylazine mixture (100 and 10 mg/kg body weight, respectively) followed by midline abdominal incision, pancreatic tissue was gently removed from the splenic lobe using saline-soaked cotton swabs, and the splenic lobe, located between the gastroduodenal junction and the spleen, was then removed, leaving the main pancreatic duct and splenic artery intact [23]. Partially pancreatectomized (Px) rats exhibited the characteristics of T2DM (random glucose levels >150 mg/dL), whereas sham-operated rats did not [24].

### 2.3. Experimental Design

A high-fat diet was prepared using a modified semi-purified AIN-93 formulation for experimental animals [25] that consisted of 42% carbohydrate, 15% protein, and 43% fat. The carbohydrate, protein, and fat sources were starch and sugar (1.35:1), casein (milk protein), and lard and corn oil (10:1; CJ Co., Seoul, Korea), respectively. The high-fat diet was supplemented with 0% (control), 0.2% (L-BC; low dosage), 0.6% (M-BC; medium dosage), and 1.8% (H-BC; high dosage) blackcurrant extracts; 0.2% metformin (positive-C); plus 1.8%, 1.6%, 1.2%, 0%, and 1.6% dextrin, specifically indigestible dextrin made from corn starch (Cuwon, Seoul, Korea). Dextrin plus blackcurrants were substituted for cellulose, and each group had an almost equivalent calorie diet (4.7 kcal/g diets). Since the blackcurrant extracts contained about 46.9% sugars and the rest was considered dietary fiber [26], indigestible dextrin was used to make an equivalent supplement. The energy content of indigestible dextrin is about 2 kcal/g [27].

Fifty Px rats were assigned randomly to the following four groups according to different diets: (1) the control group (0 mg blackcurrant extract/kg bw/day), (2) the L-BC group (100 mg blackcurrant extract/kg bw/day), (3) the M-BC group (300 mg blackcurrant extract/kg bw/day), (4) the H-BC group (900 mg blackcurrant extract/kg bw/day), and (5) the positive-C group (100 mg metformin/kg bw/day). Metformin dosage was assigned to increase arterial plasma concentrations of metformin, following a previous study [28]. AUC values of plasma metformin were 1890 ± 180, 2040 ± 360, and 2170 ± 240 μg min/mL for metformin dosages of 50, 100, and 200 mg/kg [6]. The medium dosage was chosen to compare the glucose-lowering effect of BC intake. Each group included 10 Px rats and all animals were allowed free access to water and assigned diets for eight weeks.

### 2.4. Glucose Homeostasis

Overnight-fasted serum glucose levels, food intakes, and body weights were measured weekly. Oral glucose tolerance testing (OGTT) was conducted on overnight-fasted animals at seven weeks after study commencement by orally administering 2 g glucose/kg body weight and taking tail blood samples at 0, 10, 20, 30, 40, 50, 60, 70, 80, 90, or 120 min later [29]. Serum insulin levels were measured at 0, 20, 40, 90, or 120 min after glucose administration. Areas under the curve for serum glucose and insulin were calculated using the trapezoidal rule. Three days after OGTT, intraperitoneal insulin tolerance testing (IPITT) was conducted following a 6 h fast and an intraperitoneal injection of insulin (0.75 U/kg body weight); serum glucose levels were measured every 15 min for 90 min. Serum glucose and insulin levels were analyzed with a Glucose Analyzer II (Beckman-Coulter, Palo Alto, CA, USA) and Rat Ultrasensitive Insulin Kits (Crystal Chem, Elk Grove Village, IL, USA), respectively.

### 2.5. Hyperglycemic Clamp

Two days after IPITT, catheters were surgically implanted into the right carotid artery and left jugular vein under ketamine and xylazine anesthesia. Five or six days later, hyperglycemic clamp testing was performed on free-moving and overnight-fasted rats to determine insulin secretion capacity, as described previously [24,30,31]. During the hyperglycemic clamp experiment, 5% glucose solution was infused to maintain a serum glucose level of 5.5 mM above baseline, and serum insulin levels were measured at 0, 2, 5, 10, 60, and 90 min.

After clamp testing, animals were provided with food and water ad libitum for two days and then deprived of food for 16 h. Rats were then anesthetized with ketamine/xylazine, and regular human insulin (5 U/kg body weight; Humulin; Eli Lilly, Indianapolis, IN, USA) was injected through the inferior vena cava. Ten minutes later, animals were euthanized by decapitation, and tissues were rapidly collected, frozen in liquid nitrogen, and stored at −70 °C for further experiments. Insulin resistance was determined using the homeostasis model assessment estimates of insulin resistance (HOMA-IR), which were calculated as follows: HOMA-IR = fasting insulin (µIU/mL) × fasting glucose (mM)/22.5. Serum alanine aminotransferase (ALT) and aspartate aminotransferase (AST) were measured using colorimetry kits (Asan Pharmaceutical, Seoul, Korea). TNF-α concentrations in the serum and liver lysates were determined with a radioimmunoprecipitation assay (RIPA) buffer using a Rat TNF-α ELISA kit (Invitrogen, Carlsbad, CA, USA). Lipid peroxide contents in the liver lysate were measured with a Lipid Peroxidation Assay Kit, measuring malondialdehyde (MDA) contents (Abcam, Cambridge, UK) using TBA solution.

### 2.6. Real-Time Quantitative Reverse Transcriptase-Polymerase Chain Reaction (RT-PCR)

TRIzol reagent (Life Technologies, Rockville, MD, USA) was added to each powdered hepatic sample for total RNA extraction. cDNA was synthesized from 1 μg RNA extracted from the liver sample of each rat using a superscript III reverse transcriptase kit (Life Science Technology). Equal amounts of cDNA and primers for the TNF-α gene were mixed with SYBR Green Mix (Bio-Rad, Richmond, CA, USA) in duplicate, and its amplification was measured using a real-time PCR instrument (Bio-Rad) under previously described thermal cycling conditions. The gene expression level in unknown samples was quantitated using the comparative cycle of threshold (CT) method described previously [32]. β-actin was used as an endogenous reference gene. The mRNA expression was calculated as 2^−ΔΔCT^ [32].

### 2.7. Immunohistochemistry

Five rats per group were injected with BrdU (100 µg/kg body weight) at the end of the eight-week experiment and, 6 h later, anesthetized with an intraperitoneal ketamine/xylazine injection and then sequentially perfused with saline and 4% paraformaldehyde solution (pH 7.2). Pancreases were dissected immediately after perfusion and post-fixed in 4% paraformaldehyde overnight at room temperature [33]. Two serial 5 μm paraffin-embedded tissue sections were selected from the seventh and eighth sections from the previous section to avoid counting the same islets twice when determining β-cell areas. BrdU incorporation and apoptosis were assessed by immunohistochemistry, as previously described [33]. Pancreatic β cells were identified by applying guinea pig anti-insulin and rabbit anti-glucagon antibodies to sections. β-cell areas were determined using non-overlapping images of the two insulin-stained sections at 10× with a Zeiss Axiovert microscope (Carl Zeiss Microimaging, Thornwood, NY, USA). β-cell sizes and proliferation were determined by BrdU incorporation, and apoptotic β-cell numbers in pancreatic sections were determined as previously described [33].

### 2.8. SCFA Determination

The SCFA concentrations in serum were determined using the acid alcohol methods previously described [34]. The serum was mixed with an equal volume of butanol (DUKSAN, Seoul, Korea), and HCl was added. The mixture was separated by centrifugation at 15,000 rpm for 15 min at 4 °C and filtered through a 0.45 μm microporous filter. Short-chain fatty acids in the filtrates were detected by gas chromatography (GC, Clarus 680 GAS, PerkinElmer) using an Elite-FFAP 30 m × 0.25 mm × 0.25 μm capillary column, with helium as the carrier gas at a flow rate of 1 mL/min, as previously described [34].

### 2.9. Next-Generation Sequencing of Gut Microbiomes and Fecal Microbiota Analysis

Gut microbiome compositions were determined using feces samples by metagenome sequencing with next-generation sequencing. Bacterial DNA were extracted from individual feces samples using a Power Water DNA Isolation Kit (MoBio, Carlsbad, CA, USA). DNA libraries were prepared using the GS-FLX Plus emPCR Kit (454 Life Sciences, Branford, CT, USA) and amplified using 16S universal primers (V3 region) in the FastStart High Fidelity PCR System (Roche, Basel, Switzerland), as previously described [7]. Sequencing of bacterial DNA in feces was performed by Macrogen Ltd (Seoul) using a Genome Sequencer FLX Plus (454 Life Sciences), as previously reported [7].

16S amplicon sequences were processed using Mothur v.1.36. Using the Miseq standard operating procedure (SOP), bacteria taxonomies and counts were evaluated in each fecal sample. Sequences were aligned using the Silva reference alignment (v.123) included in the Mothur SOP (https://mothur.org/wiki/silva_reference_files, accessed on 14 December 2021), and taxonomies and bacterial counts of each taxonomy were determined. Operational taxonomic units (OTUs) below 10,000 reads were deleted. Principal coordinates analysis (PCoA) was conducted using the R package and the OTU-abundance table converted to relative abundance. Chao1 and Shannon indices indicating α-diversity were calculated using the Mothur summary.single subroutine.

### 2.10. Gut Microbiota Metabolism Predicted by PICRUSt2 Pipeline Analysis

Metabolic functions of gut microbiota were predicted from 16S rRNA gene sequences of fecal bacteria using PICRUSt2, as previously described. Predicted metabolic profiles of Kyoto Encyclopedia of Genes and Genomes (KEGG) Orthologues (KO) were mapped using KEGG mapper (https://www.genome.jp/kegg/tool/map_pathway1.html, accessed on 7 January 2021) [34]. KEGG is a database designed to facilitate the understanding of the basic biological system from genomic, chemical, and molecular data. In the present study, genomic data of gut microbiota was used to explore biological differences among the groups. Abundances of mapped KOs were used to draw a heatmap with R using the heatmap.2 package.

### 2.11. Statistical Analyses

Results were expressed as means ± standard deviations and the statistical analysis was performed using SAS version 9.1 (SAS Institute, Cary, NC, USA). One-way analysis of variance (ANOVA) was used to determine the significance of differences between the control, L-BC, M-BC, H-BC, and positive-C groups. Post hoc Tukey’s tests were used to determine the significance of differences between the main effects in these groups. Statistical significance was accepted for *p* values < 0.05.

## 3. Results

### 3.1. Cyanidin 3-O-Rutinoside and Delphinidin 3-O-Rutinoside Levels in Aqueous Blackcurrant Extract

The concentrations of cyanidin 3-*O*-rutinoside and delphinidin 3-*O*-rutinoside in powdered aqueous blackcurrant extract as determined by HPLC and using standard plots (r^2^ > 0.999; Supplemental Figure S1) were 11.0 ± 0.02 and 12.3 ± 0.04 mg/g extract powder (*n* = 3). Blackcurrant extract contained 43.4 ± 0.07 mg total anthocyanins/g extract (*n* = 3).

### 3.2. Energy Metabolism

Body-weight gain over the eight-week experimental period was higher in the control group than in the positive-C group, and the M-BC and H-BC groups had the lowest body-weight gains (Table 1). Caloric intake in the control group was significantly greater than in the positive-C group, and BC intake did not affect caloric intake as compared with the control group (Table 1). Mean body-weight difference over the experimental period was reduced by BC, although caloric intakes in the BC and control groups were non-significantly different. However, food intake was lower in the positive-C group than in the control group, and water intakes were lower in the BC and positive-C groups than in the control group (Table 1). These observations indicated that BC intake reduced body weight regardless of glucose homeostasis.

Epididymal and retroperitoneal fat masses (visceral fat mass) were higher in the control group than the positive-C group (Table 1). M-BC intake decreased both epididymal and retroperitoneal fat masses but the levels observed in the positive-C group did not decrease (Table 1). Visceral fat mass (epididymal plus retroperitoneal fat mass) was lower in the M-BC and H-BC groups than in the control group and lowest in the positive-C group (Table 1).

### 3.3. Glucose Metabolism

**Figure 1:**
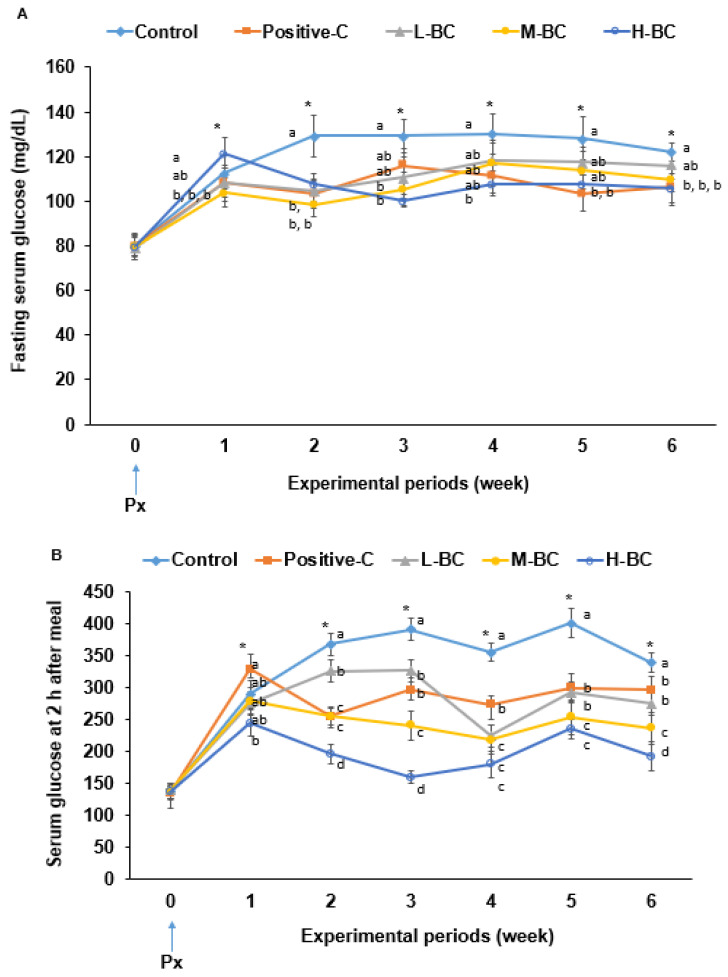
Changes of serum glucose concentrations after fasting and at 2 h post-ingestion over the eight-week experimental period. Partially pancreatectomized (Px) rats were fed a high-fat diet supplemented with 0% (control), 0.2% (L-BC; low dosage), 0.6% (M-BC; medium dosage), and 1.8% (H-BC; high dosage) blackcurrant extracts; 0.2% metformin (positive-C); plus 1.8%, 1.6%, 1.2%, 0%, and 1.6% indigestible dextrin over eight weeks. (**A**) Serum glucose concentrations in the fasted state each week. (**B**) Serum glucose concentrations in the 2 h postprandial state each week. Each dot and error bar represents a mean and standard deviation, respectively (*n* = 10). *^,a,b,c,d^ Significantly different among the groups in each week at *p* < 0.05.

Changes in overnight-fasted serum glucose concentrations in the control group were greater than in the positive-C group from experiment week 2 (EW2) to experiment week 6 (EW6) (Figure 1A). BC and metformin intake lowered overnight-fasting serum glucose concentrations compared to the control group, and their concentrations in the M-BC and H-BC groups were similar to those in the positive-C group (*p* < 0.05; Figure 1A). At 2 h post-ingestion, serum glucose concentrations were higher in the control group than in the positive-C group from EW2 to EW6 (*p* < 0.05; Figure 1B). Postprandial serum glucose concentrations were similar in the L-BC and the positive-C groups at EW3–EW6, and they were much lower in the H-BC group than the positive-C group (*p* < 0.05; Figure 1B).

Serum insulin concentrations in the fasted state were similar in the control and positive-C groups, and BC intake lowered serum insulin levels (Table 2), which were lowest in the M-BC group. Postprandial serum insulin concentrations were significantly higher in the control group than in the other four groups (Table 2). HOMA-IR (an insulin resistance index) was highest in the control group and lowest in the M-BC group (Table 2).

**Figure 2:**
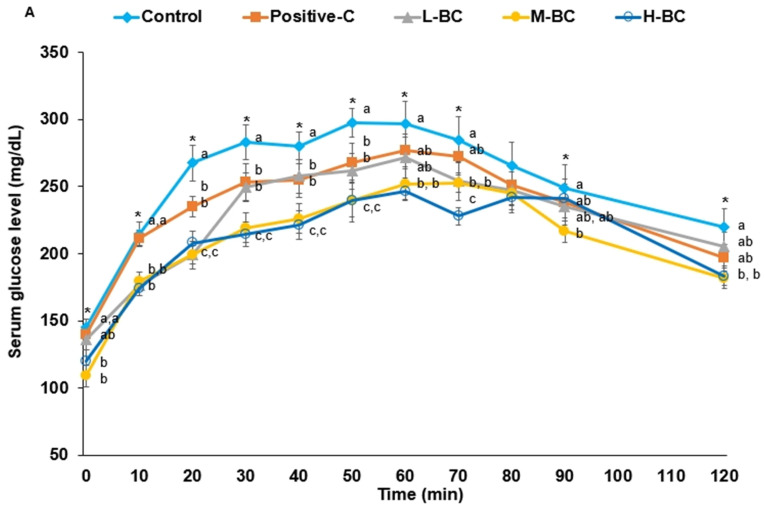

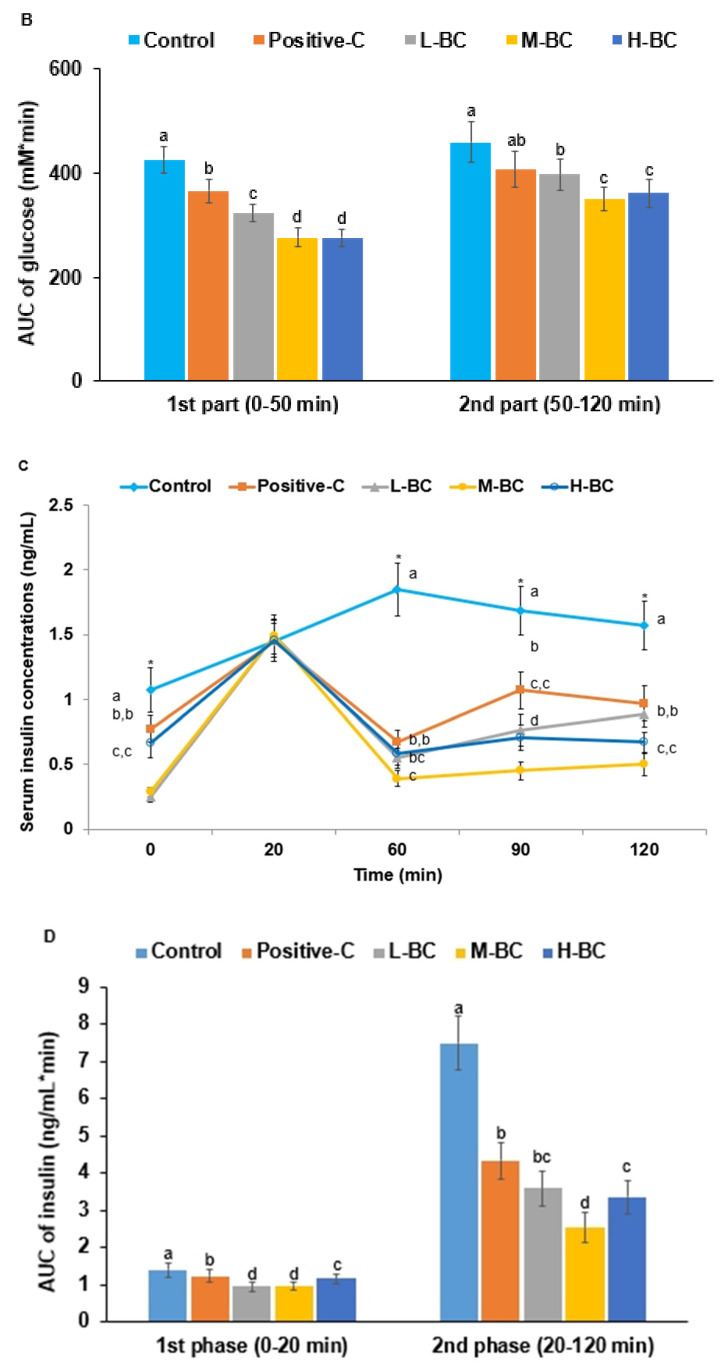
Serum glucose and insulin levels and areas under the curve (AUCs) of serum glucose and insulin concentrations during oral glucose tolerance testing (OGTT). Partially pancreatectomized (Px) rats were fed a high-fat diet supplemented with 0% (control), 0.2% (L-BC; low dosage), 0.6% (M-BC; medium dosage), and 1.8% (H-BC; high dosage) blackcurrant extracts; 0.2% metformin (positive-C); plus 1.8%, 1.6%, 1.2%, 0%, and 1.6% indigestible dextrin. After seven weeks of the assigned treatments, serum glucose levels were measured at 0, 10, 20, 30, 40, 50, 60, 70, 80, 90, and 120 min after fasting rats were administered 2 g of glucose/kg body weight orally (**A**). Areas under the curve for serum glucose concentrations were calculated from 0 to 50 min and 50 to 120 min during OGTT (**B**). Serum insulin concentrations were determined at 0, 20, 40, 90, and 120 min during OGTT (**C**). The AUC of serum insulin concentrations was calculated during the first 20 min and from 20 to 120 min (**D**). Dots/bars and error bars represent means and standard deviations, respectively (*n* = 10). * Significantly different among the groups at *p* < 0.05. ^a,b,c,d^ Different letters on bars or lines indicate significant differences among the groups at *p* < 0.05.

OGTT results showed that peak serum glucose concentrations at 50 to 60 min were highest in the control group and, in descending order, glucose concentrations followed the pattern control > positive-C = L-BC > M-BC = H-BC (Figure 2A). The time of peak serum glucose concentrations was delayed in all groups, especially in the control group, suggesting that all rats had insulin resistance and lower insulin secretion, and BC intake partially improved the delay of peak glucose. A delay in peak serum glucose concentration indicates a decrease in insulin sensitivity and secretion, and the rats in the control group had the greatest impairment of glucose homeostasis [35]. After peaking, serum glucose concentrations decreased in all groups in the order control > L-BC > positive-C > M-BC = H-BC (Figure 2A). The AUC of serum glucose 0 to 50 min after glucose administration was higher in the control group than in the positive-C group and was reduced in the order of L-BC > M-BC = H-BC (Figure 2B). BC also similarly reduced the AUCs of serum glucose from 60 to 120 min, although decreases were more marked between 0 and 50 min (Figure 2B). OGTT results in the M-BC and H-BC groups were similar.

Serum insulin concentrations rose until 60 min in the control group, but in the positive-C and BC groups they markedly increased until 20 min and then decreased (Figure 2C). From 0 to 20 min, serum insulin concentrations were higher in the control than in the BC group but were non-significantly different in the control and positive-C groups (Figure 2D). The AUCs of serum insulin concentrations from 30 to 120 min followed similar patterns as those from 0 to 20 min. However, the AUC of serum insulin concentration of the control group was much larger than that of other groups (Figure 2D), which indicated that insulin resistance in the hyperglycemic state was much greater in the control group than in the other four groups, and that insulin secretion was not regulated in the control group.

OMTT and OGTT results regarding changes in serum glucose concentrations were similar (Supplemental Figure S2A). During OMTT, serum glucose concentrations were much higher in the control group than in positive-C and BC groups. Serum glucose concentrations were similar in the L-BC, positive-C, and M-BC groups, and serum glucose concentrations were lower in the H-BC group than in the L-BC group (Supplemental Figure S2A). The AUC of serum glucose concentrations from 0 to 50 min was much higher in the control group than in the other four groups and values in the M-BC, H-BC, and positive-C groups were similar (Supplemental Figure S2B). AUC patterns from 0 to 50 min and 50 to 120 min were similar, and AUCs of serum glucose from 50 to 120 min in the H-BC and positive-C groups were also similar (Supplemental Figure S2B).

### 3.4. Insulin Tolerance

**Figure 3:**
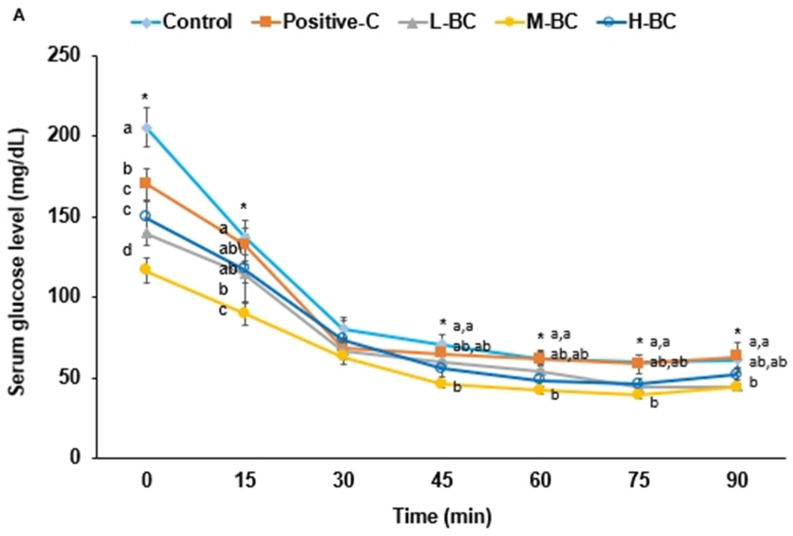

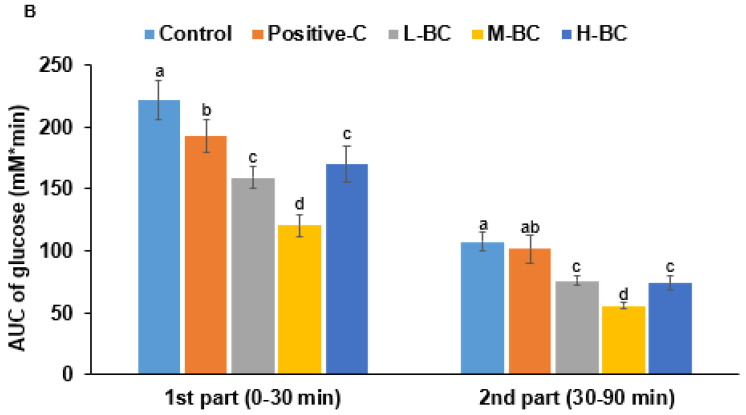
Changes and area under the curve (AUC) of serum glucose concentrations during intraperitoneal insulin tolerance testing (IPITT). Partially pancreatectomized (Px) rats were fed a high-fat diet supplemented with 0% (control), 0.2% (L-BC; low dosage), 0.6% (M-BC; medium dosage), and 1.8% (H-BC; high dosage) blackcurrant extracts; 0.2% metformin (positive-C); plus 1.8%, 1.6%, 1.2%, 0%, and 1.6% indigestible dextrin. After 6 h of food deprivation at two days after OGTT, IPITT was conducted by intraperitoneal (IP) injection of 0.75 IU insulin/kg body weight followed by measurement of serum glucose concentrations in tail blood every 15 min for 90 min. (**A**). The AUC for serum glucose during the first 0 to 30 min and 30 to 90 min (**B**). Each dot/ bar and error bar represents a mean and standard deviation, respectively (*n* = 10). * Significantly different among the groups (*p* < 0.05). ^a,b,c,d^ Different letters on bars or lines indicate significant differences among the groups at *p* < 0.05.

During IPITT, serum glucose concentrations responded to injected insulin in all groups. Serum glucose concentrations after a 6 h fast were much higher in the control group than in the other groups and followed the order control > positive-C > L-BC > H-BC > M-BC (Figure 3A). Serum glucose concentrations decreased from 30 to 45 min after insulin injection, and at 45 to 90 min were lower in the M-BC group than in the control group (Figure 3A). The AUCs of serum glucose concentrations from 0 to 30 min and 30 to 90 min after insulin injection were lowest in the M-BC group and highest in the control group (Figure 3B).

### 3.5. Hyperglycemic Clamp

**Figure 4:**
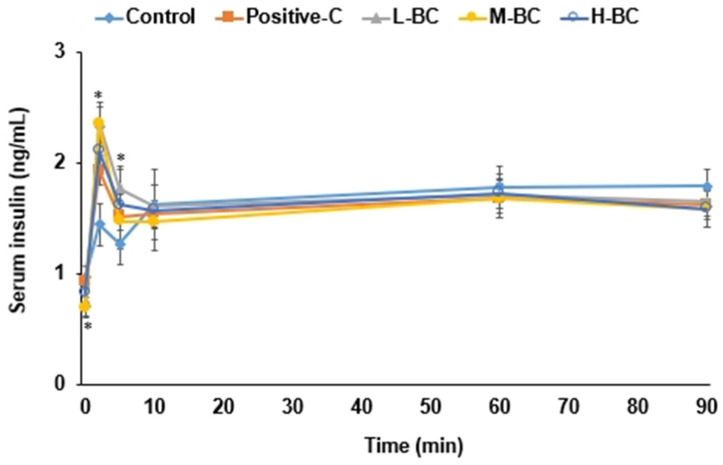
Insulin secretion during the hyperglycemic clamp. Partially pancreatectomized (Px) rats were fed a high-fat diet supplemented with 0% (control), 0.2% (L-BC; low dosage), 0.6% (M-BC; medium dosage), and 1.8% (H-BC; high dosage) blackcurrant extracts; 0.2% metformin (positive-C); plus 1.8%, 1.6%, 1.2%, 0%, and 1.6% indigestible dextrin. Hyperglycemic clamp testing was conducted on conscious, free-moving, or overnight-fasted rats to measure glucose-stimulated insulin secretion at the end of the eight-week experimental period. During a hyperglycemic clamp, exogenous glucose was infused into a jugular vein to produce serum glucose levels ~5.5 mM greater than in the fasting state, then blood was collected at 0, 2, 5, 10, 30, 60, and 90 min after infusion and serum insulin levels were measured. Each dot and error bar represents a mean and standard deviation, respectively (*n* = 10). * Significantly different among the groups at *p* < 0.05.

Serum insulin levels were measured at a serum glucose concentration of ~100 mg/dL above baseline, and these elevated levels were achieved by infusing exogenous glucose into a jugular vein. Serum insulin concentrations from 0 to 10 min after infusing glucose into the jugular vein were much lower in the control group than in the other four groups but were similar from 30 to 90 min in all groups (Figure 4, Table 2). Glucose infusion rates required to maintain serum glucose concentrations at 100 mg/dL above baseline were lower in the control group than in the other groups (Table 2). Furthermore, glucose infusion rates were higher in the M-BC and H-BC groups than in the positive-C group (Table 2). Insulin sensitivity, which was calculated using serum glucose and insulin concentrations [30], from 60 to 90 min was lower in the control group than in the other groups, and the M-BC and H-BC groups exhibited greater insulin sensitivity in the hyperglycemic state than the positive-C group (Table 2). HOMA-IR was much higher in the control group than in the other groups and was lower in the M-BC group than in the positive-C group (Table 2).

### 3.6. Pancreatic β-Cell Mass, Proliferation, and Apoptosis

Pancreatic β-cell areas, calculated by multiplying β-cell numbers by mean cell sizes, were lower in the control group than in the positive-C and H-BC groups (Table 3). Mean β-cell size was larger in the control group than in the other groups, which followed in the order of M-BC > H-BC > positive-C, indicating that insulin resistance was reduced to protect β cells from exhaustion in the M-BC, H-BC, and positive-C groups (Table 3). Total β-cell mass was calculated by multiplying pancreatic β-cell areas in the section by pancreas weights and it was found to be lowest in the control group and highest in the positive-C and H-BC groups (Table 3).

β-cell numbers are determined by the balance between β-cell proliferation and apoptosis. β-cell proliferation was lower in the control group than in the positive-C or H-BC groups (Table 3) and β-cell apoptosis was highest in the control group and lowest in the H-BC group (Table 3). These findings show that H-BC and metformin increased β-cell mass by increasing proliferation and decreasing apoptosis.

Total β-cell mass was influenced more by apoptosis than by proliferation, and β-cell apoptosis may have been regulated by oxidative stress and inflammation. Lipid peroxide contents in the islets were highest in the control group, whereas BC administration dose-dependently reduced lipid peroxide contents, which were lower in the M-BC and H-BC groups than in the positive-C group (Table 3). TNF-α mRNA expression was much higher in the control group than in the other groups and was lowest in the H-BC and positive-C groups (Table 3).

### 3.7. Liver Metabolism

The liver is a vital organ in terms of maintaining glucose homeostasis. Serum TNF-α concentrations were much higher in the control group than in the positive-C group but lower in the L-BC, positive-C, and M-BC groups and lowest in the H-BC group (Table 4). Hepatic triglyceride storage was greater in controls than in the positive-C and BC groups and lowest in the M-BC and positive-C groups. Hepatic TNF-α mRNA expressions followed a pattern similar to serum TNF-α concentrations (Table 4). Hepatic MDA contents, representing lipid peroxide contents, were higher in the controls than in the positive-C and BC groups. These results show that the liver was exposed to higher inflammatory and oxidative stresses in control rats than in rats in the positive-C, M-BC, and H-BC groups. Furthermore, elevated serum ALT and AST concentrations in the control group indicated more hepatic damage than in the positive-C and BC groups (Table 4).

### 3.8. Serum SCFA Concentrations and Gut Microbiome

**Figure 5:**
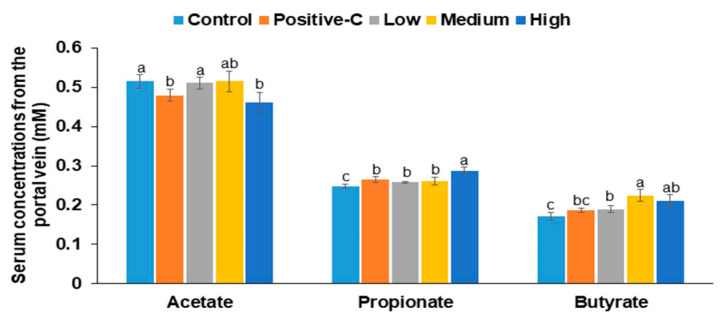
Serum short-chain fatty acid concentrations from the portal vein. Partially pancreatectomized (Px) rats were fed a high-fat diet supplemented with 0% (control), 0.2% (L-BC; low dosage), 0.6% (M-BC; medium dosage), and 1.8% (H-BC; high dosage) blackcurrant extracts; 0.2% metformin (positive-C); plus 1.8%, 1.6%, 1.2%, 0%, and 1.6% indigestible dextrin. At the end of the experiment, blood was collected from the portal vein, and serum was separated. Acetate, propionate, and butyrate concentrations were measured by gas chromatography. Each bar and error bar represents a mean and standard deviation, respectively (*n* = 10). ^a,b,c^ Different letters on bars indicate significant differences among the groups at *p* < 0.05.

Serum acetate concentrations were lower in the positive-C and H-BC groups than the in the control group (Figure 5). However, serum propionate and butyrate concentrations were lower in the control group than in the other groups. M-BC increased serum propionate concentrations, and H-BC elevated serum butyrate concentrations the most (Figure 5).

**Figure 6:**
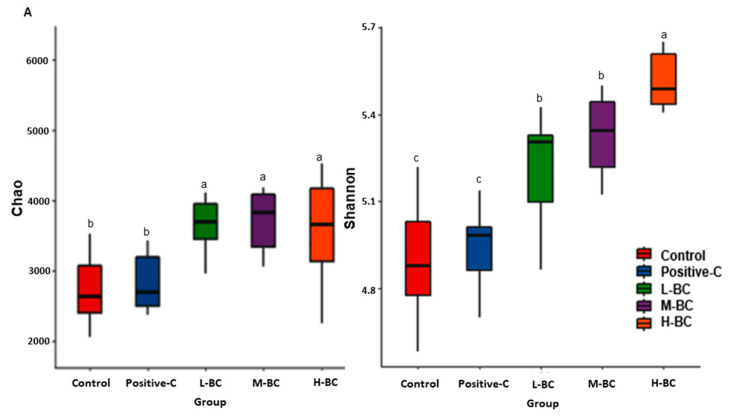

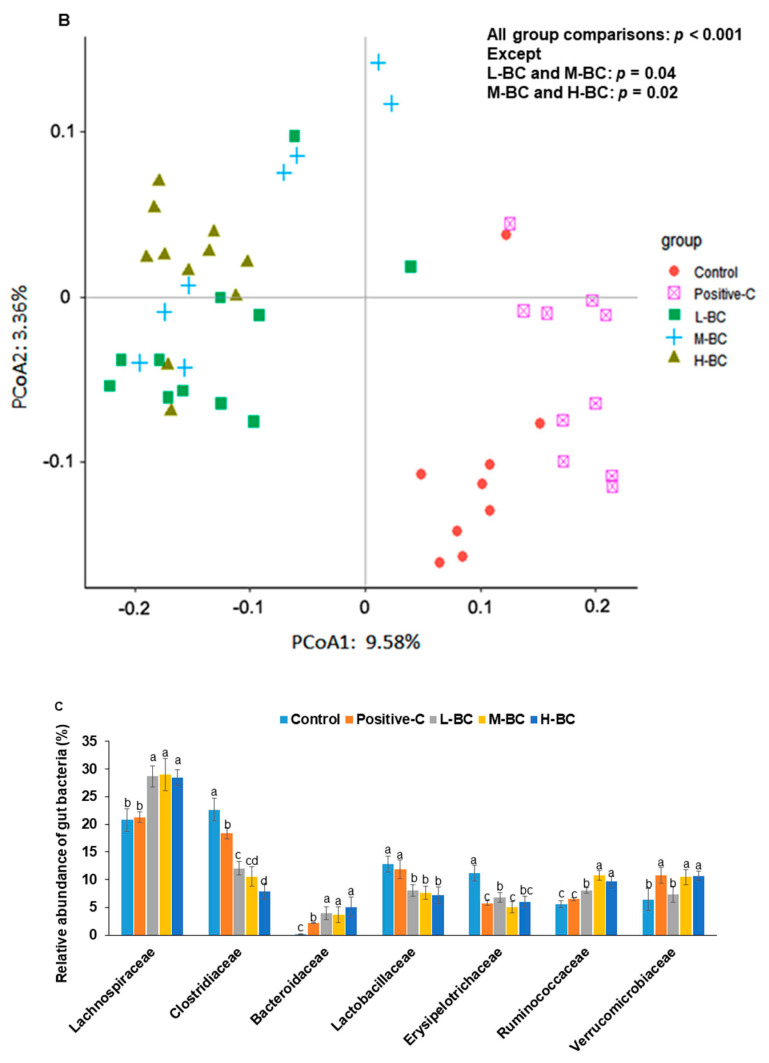

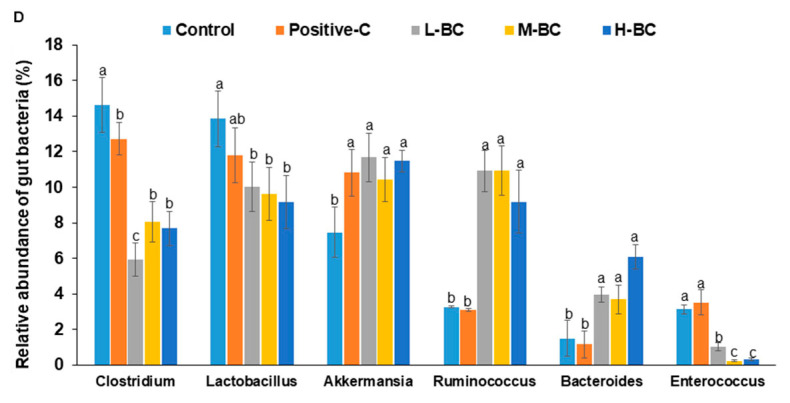
Profiles of the gut microbiome as determined by NGS analysis of bacterial DNA from the feces of the cecum. Partially pancreatectomized (Px) rats were fed a high-fat diet supplemented with 0% (control), 0.2% (L-BC; low dosage), 0.6% (M-BC; medium dosage), and 1.8% (H-BC; high dosage) blackcurrant extracts; 0.2% metformin (positive-C); plus 1.8%, 1.6%, 1.2%, 0%, and 1.6% indigestible dextrin. (**A**) α-diversity of gut microbiomes representing bacteria richness measured by Chao1 and Shannon index. (**B**) β-diversity of bacterial communities separated by principal coordinate analysis (PCoA). (**C**,**D**) Proportions of taxonomic assignments of major bacteria at the family (**C**) and genus (**D**) levels for gut microbiomes. Each bar/dot and error bar represents a mean and standard deviation, respectively (*n* = 10). ^a,b,c,d^ Different letters on bars indicate significant differences among the groups at *p* < 0.05.

α-diversity is a quantitative assessment of the number of bacterial species present and in the present study it was determined using chao1 and Shannon indexes. These two indexes were similar in the control and the positive-C groups but higher in the BC groups than in the control group (Figure 6A). According to Shannon indexes, α-diversity dose-dependently increased with BC (*p* < 0.05; Figure 6A). Principle coordinate analysis (PCoA) showed β-diversity of the gut microbiome: fecal bacterial communities formed well-separated clusters in the five study groups (Figure 6B). The control group was clearly separated from the positive-C and BC groups (*p* < 0.001). BC groups formed a large cluster (*p* < 0.001), though each group was clustered into subgroups according to the dosages of BC (Figure 6B). These results indicate that α- and β-diversities were improved in the BC groups compared to the control group. However, those of the control and positive-C groups were not significantly different.

Analysis of molecular variance (AMOVA) showed that community compositions of gut microbiota were significantly different in the study groups (*p* < 0.001) and significantly different in the control and BC groups (*p* < 0.001), but not between the control and positive-C groups. Furthermore, bacterial distributions differed at the family and genus levels (Figure 6C,D). At the family level, the primary bacteria were Clostridaceae, Lachnospiraceae, Bacteroidaceae, Coriobacteriaceae, Lactobacillaceae, and Ruminococcaceae (Figure 6C). The relative abundances of Lachnospiraceae and Ruminococcaceae were much lower, but those of Clostridaceae, Lactobacillaceae, and Coriobacteriaceae were higher in the control and positive-C groups than in the BC groups (Figure 6C). At the genus level, the relative abundances of *Lactobacillus* and *Clostridium* were higher, and those of *Bacteroides, Ruminococcus,* and *Akkermensia* were lower in the control group than in the BC groups (Figure 6D).

### 3.9. Metabolic Activities of Intestinal Bacteria as Determined by PICRUSt2 Analysis

**Figure 7:**
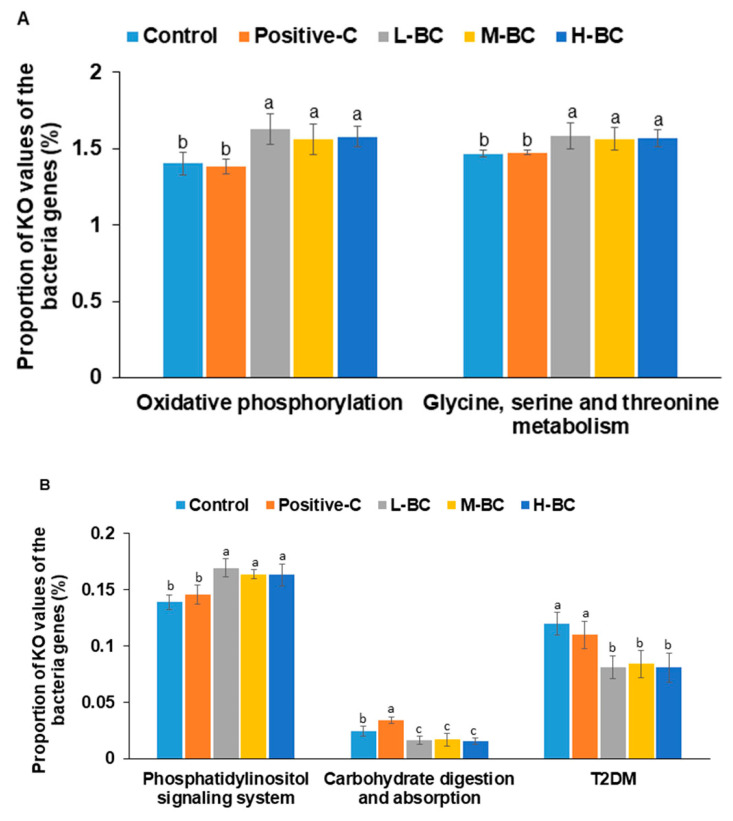
Prediction of gene function of the fecal bacteria by PICRUSt2. Partially pancreatectomized (Px) rats were fed a high-fat diet supplemented with 0% (control), 0.2% (L-BC; low dosage), 0.6% (M-BC; medium dosage), and 1.8% (H-BC; high dosage) blackcurrant extracts; 0.2% metformin (positive-C); plus 1.8%, 1.6%, 1.2%, 0%, and 1.6% indigestible dextrin for eight weeks. KO, Kyoto Encyclopedia of Genes and Genomes Orthologues. The proportion of KO values of the fecal bacteria genes in (**A**) oxidative phosphorylation and glycine, serine, and threonine metabolism. (**B**) Phosphatidylinositol signaling system, carbohydrate digestion and absorption, and type 2 diabetes (T2DM). Each bar represents the proportion of KO values of the designated metabolism in fecal bacteria genes of each group (*n* = 10). ^a,b,c^ Different letters on bars indicate significant differences among the groups at *p* < 0.05.

Figure 7 presents the abundances of KEGG orthologues involved in starch and sucrose, glycine, serine, threonine, and inositol phosphate metabolism, as well as the oxidative phosphorylation of the gut microbiome (*p* < 0.00001 in all metabolisms). The relative abundances of fecal bacteria involved in energy metabolism-associated oxidative phosphorylation was higher in the BC groups than in the control and positive-C groups, which may have been associated with lower total visceral fat levels in the BC groups (Figure 7A). The relative abundances of fecal bacteria involved in glycine, serine, and threonine metabolism and phosphatidylinositol signaling, which are involved in the energy and glucose metabolism of the gut microbiome, were greater in BC groups than in the control and positive-C groups (*p* < 0.00001; Figure 7A). The significant bacteria involved in glycine, serine, and threonine metabolism and oxidative phosphorylation were primarily and commonly composed of *Akkermentia*, *Blautia*, *SMB53*, *Clostridium*, *Lactobacillus*, *Dorea*, and *Ruminococcus*. In contrast, the relative abundances of fecal bacteria involved in carbohydrate digestion and absorption were significantly lower in BC groups but were higher in the positive-C group than in the control group (*p* < 0.00001; Figure 7B). Integration of insulin sensitivity and insulin secretion signaling showed that the relative abundances of fecal bacteria associated with T2DM risk were lower in the BC groups than in the control group (*p* < 0.00001; Figure 7B).

## 4. Discussion

Partially pancreatectomized rats fed a high-fat diet have been shown to increase insulin resistance in the absence of hyperinsulinemia and obesity [35]. This research demonstrated for the first time that powdered aqueous BC extract intake (300 to 500 mg/kg bw/day; human equivalent dosage—about 1 g) enhanced insulin sensitivity and partially normalized insulin secretion when serum glucose concentrations were suddenly increased in a non-obese insulin insufficient T2DM animal model. Furthermore, pancreatic β-cell mass was greatly increased by powdered aqueous BC extract administration, and this was primarily related to less apoptosis, inflammation, and oxidative stress associated with gut microbiota changes. Streptozotocin and alloxan administration can induce hyperglycemia by increasing β-cell death in non-obese rats. However, their administration increases β-cell death through elevated reactive oxygen species, removal of which can reduce diabetic severity during treatment [36,37]. They also induce neuronal cell death [36]. The non-surgical model cannot explain the modulation of β-cell function and mass with insulin resistance. The present study examined the antidiabetic activity and action mechanism of blackcurrant extracts in partially pancreatectomized rats with similar characteristics to Asian T2DM.

Blackcurrant berries have various biological properties associated with high anthocyanin contents, the latter consisting primarily of delphinidin 3-*O*-rutinosides and cyanidin 3-*O*-rutinosides [38], which was confirmed by our findings [39]. Anthocyanins exert antidiabetic effects by improving insulin resistance and insulinotropic activity through the stimulation of GLP-1 secretion in animals and humans [40]. Delphinidin 3-*O*-rutinosides act as a potent stimulator of GLP-1 secretion [41] and cyanidin 3-*O*-rutinosides also alleviate postprandial hyperglycemia by inhibiting intestinal a-amylase [42]. Thus, they are potentially effective ingredients for hyperglycemia in blackcurrant extract. However, extracts of anthocyanin-containing foods like blackcurrants must be managed at low temperatures under acidic conditions, such as in the presence of citric acid or vitamin C, since anthocyanins are sensitive to heat and require neutral or alkaline conditions [15,43]. In the present study, because blackcurrant extracts are naturally acidic, the powdered BC extract was produced at 80 °C over 2 h in the absence of vitamin C and then lyophilized.

The present study showed that M-BC or H-BC intake (0.7 and 2 g/day in human equivalents, respectively) reduced body weight and visceral fat mass and improved insulin resistance and glucose-stimulated insulin secretion, thus improving glucose homeostasis. It also reduced lipid peroxide contents in the liver and islets, which can be expected to improve hepatic insulin resistance and β-cell survival and thus prevent the deterioration of glucose homeostasis. However, L-BC (0.2 g/day) intake had a negligible effect compared to M-BC and H-BC (0.7 to 2 g/day) intake in the present study. Thus, the findings of the present study suggest that an intake of 0.7 to 2 g/day might be sufficient for antidiabetic activity. Several previous studies have demonstrated that blackcurrant extract intake attenuates insulin resistance in rats and overweight or obese individuals [44,45]. In obese or overweight individuals, an eight-day intake of blackcurrant extract rich in anthocyanins (600 mg/day blackcurrant extract including 210 mg anthocyanin) significantly improved insulin sensitivity by 22% and decreased serum CRP concentrations [44]. In another study, blackcurrant extract administration (1.5 g/day human equivalents) for eight weeks ameliorated metabolic syndrome by potentiating IRS-1 and AMPK phosphorylations, thus improving insulin sensitivity in the skeletal muscle of rats consuming high fructose diets with fructose-induced metabolic syndrome [45]. Anthocyanin-containing food extracts (2 g/day human equivalents) have also been shown to reduce insulin resistance in estrogen-deficient rats [15]. Anthocyanins demonstrate competitive inhibitory activity against α-amylases and α-glucosidases like acarbose by binding to their active sites and have shown been to lower postprandial glucose concentrations in molecular docking analyses [19]. Therefore, aqueous blackcurrant extract intake of about 1 to 2 g/day may encourage antidiabetic activity by improving insulin sensitivity and suppressing α-amylase activity.

BC intake (500 mg anthocyanin) in humans has been reported to increase serum GLP-1 concentrations at 90 min modestly and to decrease serum insulin concentrations [45,46]. GLP-1 is known to stimulate glucose-stimulated insulin secretion and β-cell mass in diabetic partially pancreatectomized rats [47]. Furthermore, blackcurrant extract intake (0.3 g/day human equivalents) reduces malondialdehyde contents by increasing antioxidant enzymes, such as superoxide dismutase, catalase, and glutathione peroxidase, indicating a reduction of oxidative stress [48]. We also demonstrated that TNF-α expression and MDA contents in islets were higher in the control group than in the positive-C group and that BC administration decreased them to the same levels as the positive-C group. These results suggest that increased oxidative stress and inflammation in islets impaired β-cell function and mass in the control group and that BC administration markedly suppressed these effects to promote antidiabetic activity.

Blackcurrant intake is known to modify the gut microbiome composition and to impact glucose metabolism directly [49]. The present study shows that α-diversity of gut microbiomes, as determined using Chao1 and Shannon indices, dose-dependently increased in the BC groups compared with the control and positive-C groups. Lower α-diversity is generally accepted as a characteristic of T2DM, which is consistent with our results [50]. Moreover, β-diversity showed a clear separation of gut bacteria among all groups. The gut microbiota of the BC groups were clearly clustered apart from those of the control and positive-C groups, and that of the positive-C group was also separated from that of the control group. These results demonstrate that BC intake changes gut microbiota, which may be a factor in the alleviation of hyperglycemia. In control rats, the composition of Lachnospiraceae was lower, but that of Clostridacea was higher than in the positive-C and BC groups. Furthermore, Lactobacillaceae composition was lower in the BC and positive-C groups than in the control group, but *Akkermansia muciniphila* contents were much higher in the BC group than the control. Intake of anthocyanin-rich foods, such as blackcurrants and blackberries, enriches *Akkermansia muciniphila*, which is consistent with our findings [51,52]. Fecal Bacteroidetes levels are significantly decreased in T2DM patients, similar to the findings of the present study [53]. The present study showed that BC intake increased serum propionate and butyrate concentrations, but not that of acetate, compared to the control. Previous studies have demonstrated that butyrate administration significantly increases levels of the butyrate receptor G-protein-coupled receptor 43 to elevate glycogen storage by upregulating glucose transporter-2 and activating PKB→GSK3 in T2DM mice, HepG2 cells, and type 2 diabetic patients [53,54]. Consistent with serum SCFA concentrations, PICRUSt2 analysis revealed that BC intake predicted increased butyric acid production. Furthermore, it predicted the potential to reduce carbohydrate digestion and absorption and downregulate phosphatidylinositol signaling, indicating improved glucose metabolism. However, reports of *Bifidobacterium* and *Lactobacillus* elevation in the gut are inconsistent with regard to antidiabetic effects, including the present study [55]. Thus, serum SCFA concentrations and a-diversity may be better indicators to show the improvement of hyperglycemia than the relative abundance of individual gut bacteria.

## 5. Conclusions

BC intake decreased oxidative stress and inflammation and suppressed apoptosis but increased β-cell mass by increasing β-cell counts. It would appear that BC acted indirectly by changing the composition of gut microbiota and directly through the activities of its constituents. Furthermore, BC increased α-diversity, reduced carbohydrate digestion and absorption, increased oxidative phosphorylation and phosphatidylinositol signaling, and promoted glucose metabolism. The antidiabetic effects of BC and effects on the microbiome diversity were both dose-dependent, suggesting that the effects could be linked. In conclusion, BC exerted T2DM-associated effects by improving insulin sensitivity and glucose-stimulated glucose uptake and alleviated T2DM symptoms in our non-obese, insulin-deficient animal model.

## Figures and Tables

**Table 1:** Body weight, food intake, and visceral fat mass after the eight-week intervention.

	Px-Control (*n* = 10)	Positive-C (*n* = 10)	L-BC (*n* = 10)	M-BC (*n* = 10)	H-BC (*n* = 10)
Body weight (g)	378 ± 14 ^a^	357 ± 12 ^b^	375 ± 7.7 ^a^	356 ± 11 ^b^	362 ± 13 ^b^
Weight gain during the eight-week intervention (g)	150 ± 11 ^a^	135 ± 8.2 ^b^	142 ± 9.1 ^a,b^	137 ± 11 ^b^	140 ± 7.8 ^b^
Epididymal fat pads (g)	7.8 ± 0.6 ^a^	6.0 ± 0.4 ^c^	8.1 ± 0.5 ^a^	6.7 ± 0.5 ^b^	7.2 ± 0.5 ^b^
Retroperitoneal fat (g)	9.5 ± 0.9 ^a^	7.1 ± 0.7 ^c^	9.2 ± 0.5 ^a^	7.6 ± 0.5 ^c^	8.5 ± 0.4 ^b^
Visceral fat mass (g)	17.4 ± 1.46 ^a^	13.1 ± 1.08 ^c^	17.7 ± 0.78 ^a^	14.2 ± 1.04 ^c^	15.8 ± 0.97 ^b^
Caloric intake (kcal/day)	88.2 ± 5.6 ^a^	75.6 ± 3.3 ^b^	92.3 ± 4.4 ^a^	89.8 ± 7.1 ^a^	88.1 ± 4.3 ^a^
Blackberry extract intake (mg/kg/bw)	-	-	108 ± 5.2	322 ± 25	931 ± 45
Metformin intake (mg/kg/bw)		92.3 ± 4.0			
Water intake (mL/day)	47.0 ± 3.0 ^a^	39.4 ± 3.1 ^b^	38.7 ± 2.9 ^b^	33.2 ± 2.4 ^c^	30.8 ± 2.5 ^c^

Partially pancreatectomized (Px) rats were fed a high-fat diet supplemented with 0% (control), 0.2% (L-BC; low dosage), 0.6% (M-BC; medium dosage), and 1.8% (H-BC; high dosage) blackcurrant extracts; 0.2% metformin (positive-C); plus 1.8%, 1.6%, 1.2%, 0%, and 1.6% dextrin, specifically indigestible dextrin, over eight weeks. Each value represents a mean ± SD (*n* = 10). ^a,b,c^ Different superscript letters on values indicate significant differences (*p* < 0.05).

**Table 2:** Serum glucose and insulin concentrations, glucose infusion rats, and insulin sensitivity during the hyperglycemic clamp.

	Px-Control (*n* = 10)	Positive-C (*n* = 10)	L-BC (*n* = 10)	M-BC (*n* = 10)	H-BC (*n* = 10)
Serum glucose at fasting state (mg/dL)	128 ± 4.3 ^a^	104 ± 8.1 ^c^	118 ± 6.2 ^b^	114 ± 8.4 ^b^	108 ± 5.7 ^c^
Serum insulin at fasting state (mg/dL)	0.97 ± 0.10 ^a^	0.92 ± 0.07 ^a^	0.75 ± 0.13 ^b,c^	0.67 ± 0.09 ^c^	0.82 ± 0.07 ^b^
Serum glucose at 2 h postprandial state (mg/dL)	356 ± 14 ^a^	239 ± 15 ^b^	225 ± 18 ^b,c^	209 ± 22 ^c^	181 ± 21 ^d^
Serum insulin at 2 h postprandial state (mg/dL)	1.89 ± 0.15 ^a^	1.62 ± 0.14 ^b^	1.65 ± 0.14 ^b^	1.58 ± 0.16 ^b^	1.59 ± 0.16 ^b^
HOMA-IR	9.0 ± 0.8 ^a^	6.9 ± 0.4 ^b^	6.4 ± 0.7 ^b^	5.6 ± 0.6 ^c^	6.4 ± 0.5 ^b^
Serum insulin at 2 to 10 min (ng/mL)	1.36 ± 0.22 ^b^	1.72 ± 0.13 ^a^	1.96 ± 0.27 ^a^	1.91 ± 0.30 ^a^	1.86 ± 0.26 ^a^
Serum insulin at 60 to 90 min (ng/mL)	1.79 ± 0.17	1.65 ± 0.16	1.68 ± 0.13	1.63 ± 0.17	1.66 ± 0.17
Glucose infusion rates (mg/kg bw/min)	9.1 ± 0.8 ^c^	10.2 ± 0.7 ^b^	10.5 ± 0.7 ^b^	10.9 ± 0.8 ^a,b^	11.3 ± 0.8 ^a^
Insulin sensitivity at hyperglycemic state (µmol glucose min^−1^ 100 g^−1^ per µmol insulin/L)	19.3 ± 1.8 ^c^	23.5 ± 2.2 ^b^	23.8 ± 1.8 ^b^	25.4 ± 1.8 ^a^	25.9 ± 2.2 ^a^

Partially pancreatectomized (Px) rats were fed a high-fat diet supplemented with 0% (control), 0.2% (L-BC; low dosage), 0.6% (M-BC; medium dosage), and 1.8% (H-BC; high dosage) blackcurrant extracts; 0.2% metformin (positive-C); plus 1.8%, 1.6%, 1.2%, 0%, and 1.6% indigestible dextrin. After infusion of 5% glucose solution into the jugular vein, serum glucose concentration was raised to 100 mg/dL above baseline, and serum insulin concentrations were measured at 0, 2, 5, 10, 60, and 90 min. Each value represents the mean ± standard deviation (*n* = 10). ^a,b,c,d^ Different superscript letters on the values indicate significant differences at *p* < 0.05.

**Table 3:** The β-cell mass and β-cell proliferation and apoptosis at eight-week intervention.

	Control (*n* = 5)	Positive-C (*n* = 5)	L-BC (*n* = 5)	M-BC (*n* = 5)	H-BC (*n* = 5)
β-cell area (%)	6.3 ± 0.7 ^b^	7.1 ± 0.8 ^a^	6.8 ± 0.8 ^a,b^	6.9 ± 0.8 ^a,b^	7.6 ± 0.9 ^a^
Individual β-cell size (μm^2^)	245 ± 22 ^a^	195 ± 18 ^b,c^	212 ± 20 ^b^	198 ± 22 ^b,c^	191 ± 19 ^c^
Absolute β-cell mass (mg)	23.8 ± 1.8 ^c^	31.3 ± 2.5 ^a^	26.3 ± 2.2 ^b^	28.8 ± 2.3 ^b^	32.4 ± 3.2 ^a^
BrdU^+^ cells (% BrdU^+^ cells of islets)	0.82 ± 0.09 ^b^	1.01 ± 0.09 ^a^	0.89 ± 0.10 ^b^	0.90 ± 0.10 ^b^	1.05 ± 0.11 ^a^
Apoptosis (% apoptotic bodies of islets)	0.75 ± 0.08 ^a^	0.61 ± 0.07 ^b,c^	0.66 ± 0.07 ^b^	0.65 ± 0.07 ^b^	0.58 ± 0.05 ^c^
Lipid peroxides (MDA nmol/mg protein)	3.45 ± 0.37 ^a^	2.18 ± 0.31 ^c^	2.76 ± 0.28 ^b^	2.12 ± 0.25 ^c^	2.09 ± 0.29 ^c^
Relative mRNA TNF-α expression (AU)	1.0 ± 0 ^a^	0.82 ± 0.11 ^b^	0.79 ± 0.10 ^b^	0.70 ± 0.11 ^b,c^	0.68 ± 0.10 ^c^

Partially pancreatectomized (Px) rats were fed a high-fat diet supplemented with 0% (control), 0.2% (L-BC; low dosage), 0.6% (M-BC; medium dosage), and 1.8% (H-BC; high dosage) blackcurrant extracts; 0.2% metformin (positive-C); plus 1.8%, 1.6%, 1.2%, 0%, and 1.6% indigestible dextrin. MDA, malondialdehyde; TNF, tumor-necrosis factor; AU, arbitrary unit. Each value represents the mean ± SD (*n* = 10). ^a,b,c^ Different superscript letters on the values indicate significant differences between the groups at *p* < 0.05.

**Table 4:** Triglyceride contents, inflammation, and oxidative stress indexes in the liver.

	Px-Control (*n* = 10)	Positive-C (*n* = 10)	L-BC (*n* = 10)	M-BC (*n* = 10)	H-BC (*n* = 10)
Serum TNF-α (pg/mL)	8.9 ± 0.7 ^a^	5.6 ± 0.6 ^b^	5.8 ± 0.6 ^b^	4.5 ± 0.5 ^c,d^	4.8 ± 0.6 ^c^
Hepatic TNF-α expression (AU)	1.0 ± 0.12 ^a^	0.85 ± 0.11 ^b^	0.91 ± 0.11 ^a,b^	0.81 ± 0.10 ^b^	0.80 ± 0.10 ^b^
Hepatic lipid peroxides (MDA nmol/mg protein)	3.05 ± 0.20 ^a^	1.58 ± 0.11 ^c^	2.80 ± 0.16 ^b^	1.66 ± 0.15 ^c^	1.62 ± 0.20 ^c^
Hepatic triglyceride (mg/g tissue)	47.6 ± 4.8 ^a^	32.9 ± 5.0 ^c^	43.5 ± 4.4 ^a,b^	32.6 ± 5.2 ^c^	41.9 ± 3.7 ^b^
Serum ALT (IU/L)	54.0 ± 3.2 ^a^	41.0 ± 3.6 ^c^	46.3 ± 1.7 ^b^	47.0 ± 3.5 ^b^	41.7 ± 2.2 ^c^
Serum AST (IU/L)	38.2 ± 3.8 ^a^	33.0 ± 2.6 ^b^	26.8 ± 1.8 ^c^	27.0 ± 2.0 ^c^	24.1 ± 1.7 ^d^

Partially pancreatectomized (Px) rats were fed a high-fat diet supplemented with 0% (control), 0.2% (L-BC; low dosage), 0.6% (M-BC; medium dosage), and 1.8% (H-BC; high dosage) blackcurrant extracts; 0.2% metformin (positive-C); plus 1.8%, 1.6%, 1.2%, 0%, and 1.6% indigestible dextrin. TNF, tumor-necrosis factor; MDA, malondialdehyde; AU, arbitrary unit; ALT, alanine aminotransferase; AST, aspartate aminotransferase. Each value represents the mean ± SD (*n* = 10). ^a,b,c,d^ Different superscript letters on the values indicates significant differences between the groups (*p* < 0.05).

## Data Availability

The data used in the present study are available on request from the corresponding author.

## Supplementary Materials

The following are available online at https://www.mdpi.com/article/10.3390/antiox10050756/s1, Figure S1: Delphinidin-3- rutinoside, Cyanidin-3-rutinoside contents in standards and aqueous blackcurrant extracts, Figure S2: Serum glucose levels and area under the curve (AUC) of serum glucose and insulin during oral maltose tolerance test (OMTT).
