# Supplementary material for: Aqueous Blackcurrant Extract Improves Insulin Sensitivity and Secretion and Modulates the Gut Microbiome in Non-Obese Type 2 Diabetic Rats
Source: Antioxidants (Basel). 2021 May 10;10(5):756. doi: 10.3390/antiox10050756 (PMC8150986; doi:10.3390/antiox10050756)
Supplement: Supplementary file 1 [file antioxidants-10-00756-s001.zip › antioxidants-1200186-supplementary.pdf]

Supplemental Fig. 1. Delphinidin-3-rutinoside, Cyanidin-3-rutinoside contents in standards and aqueous blackcurrant extracts

A . Standard curves of cyanidin 3-rutinoside and delphinidin 3-rutinoside.

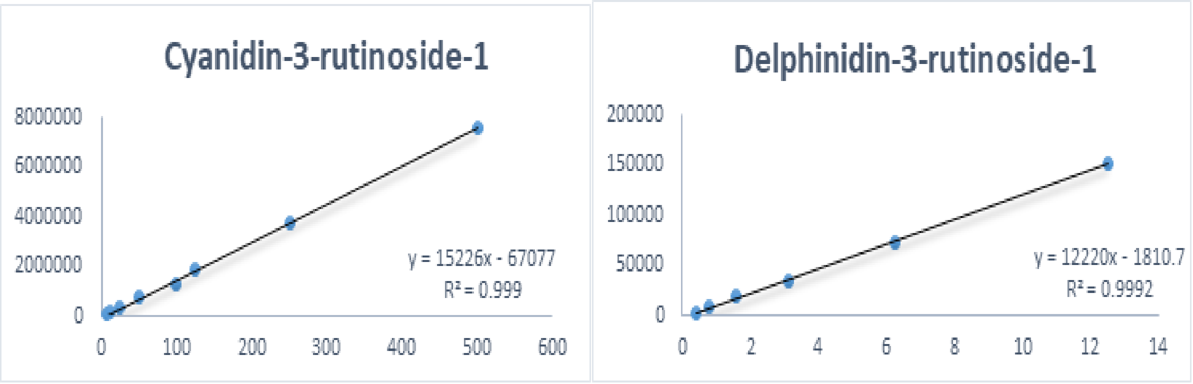

B. Chromatograms of standards and aqueous blackcurrant extract

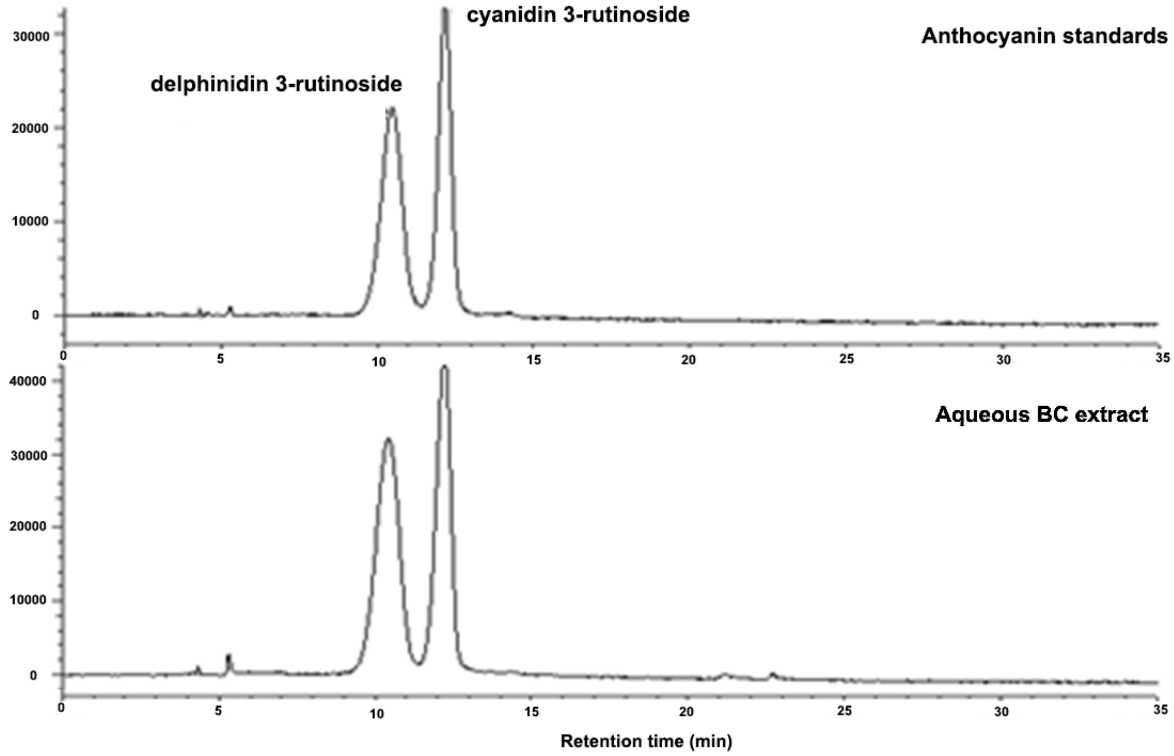

Supplemental Fig. 2. Serum glucose levels and area under the curve (AUC) of serum glucose and insulin during oral maltose tolerance test (OMTT)

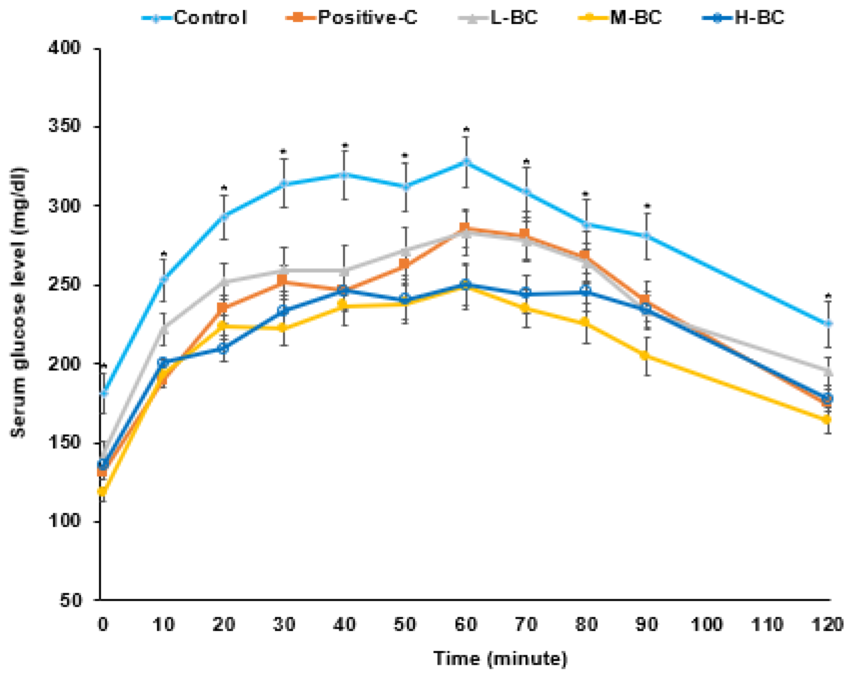

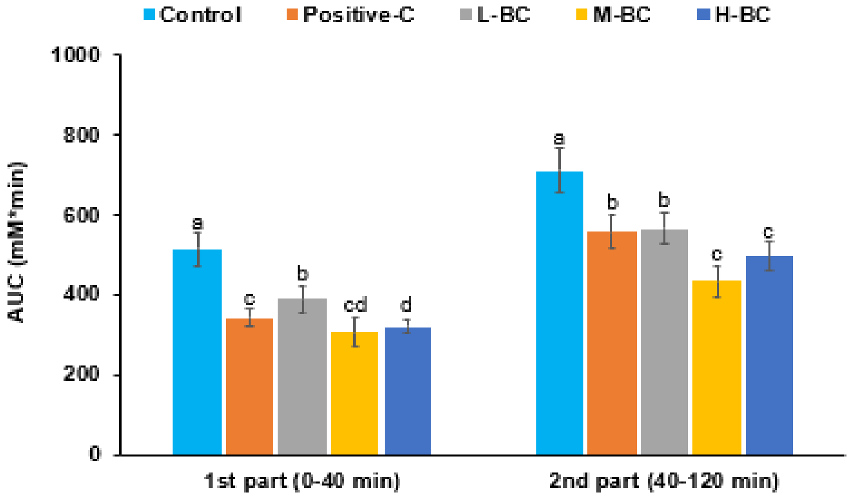

Partially pancreatectomized (Px) rats fed a high fat diet supplemented with 0% (Control), 0.2% (L-BC; low dosage), 0.6% (M-BC; medium dosage), 1.8% blackcurrant extracts (H-BC; high dosage), 0.2% metformin (positive-C) plus 1.8%, 1.6%, 1.2%, 0%, and 1.6% indigestible dextrin. At 2 days after OGTT at 7th week, the changes of serum glucose concentrations at 0, 10, 20, 30, 40, 50, 60, 70, 80, 90, 120 min after oral intake of 2 g maltose/kg bw (A) and area

under the curve (AUC) of serum glucose concentrations (B) were measured after oral intake of 2 g maltose /kg body weight.

Each dot or bar and error bar represent the means±standard deviations (n=10).

*Significantly different among the groups at $P<0.05$.

[a,b,c] Different letters on bars or lines indicate significant differences among the groups at $p < 0.05$.
